# Cyanobacteria as Nanogold Factories II: Chemical Reactivity and anti-Myocardial Infraction Properties of Customized Gold Nanoparticles Biosynthesized by *Cyanothece* sp.

**DOI:** 10.3390/md17070402

**Published:** 2019-07-08

**Authors:** Nancy S. Younis, Esam M. Bakir, Maged E. Mohamed, Nermin A. El Semary

**Affiliations:** 1 Pharmaceutical Sciences Department, College of Clinical Pharmacy, King Faisal University, Al-Ahsa 31982, Saudi Arabia; 2 Pharmacology Department, Zagazig University, Zagazig 44519, Egypt; 3 Chemistry Department, College of Science, King Faisal University, Al-Ahsa 31982, Saudi Arabia; 4 Chemistry Department, Faculty of Science, Ain Shams University, Al-bassia, Cairo 11566, Egypt; 5 Pharmacognosy Department, College of Pharmacy, Zagazig University, Zagazig 44519, Egypt; Biological Sciences Department, College of Science, King Faisal University, Al-Ahsa 31982, Saudi Arabia; 7 Botany and Microbiology Department, Faculty of Science, Helwan University, Ain Helwan, Cairo 11795, Egypt

**Keywords:** *Cyanothece* sp., Gold nanoparticles different sizes, Myocardial infarction

## Abstract

*Cyanothece* sp., a coccoid, unicellular, nitrogen-fixing and hydrogen-producing cyanobacterium, has been used in this study to biosynthesize customized gold nanoparticles under certain chemical conditions. The produced gold nanoparticles had a characteristic absorption band at 525–535 nm. Two types of gold nanoparticle, the purple and blue, were formed according to the chemical environment in which the cyanobacterium was grown. Dynamic light scattering was implemented to estimate the size of the purple and blue nanoparticles, which ranged from 80 ± 30 nm and 129 ± 40 nm in diameter, respectively. The highest scattering of laser light was recorded for the blue gold nanoparticles, which was possibly due to their larger size and higher concentration. The appearance of anodic and cathodic peaks in cyclic voltammetric scans of the blue gold nanoparticles reflected the oxidation into gold oxide, followed by the subsequent reduction into the nano metal state. The two produced forms of gold nanoparticles were used to treat isoproterenol-induced myocardial infarction in experimental rats. Both forms of nanoparticles ameliorated myocardial infarction injury, with a slight difference in their curative activity with the purple being more effective. Mechanisms that might explain the curative effect of these nanoparticles on the myocardial infarction were proposed. The morphological, physiological, and biochemical attributes of the *Cyanothece* sp. cyanobacterium were fundamental for the successful production of “tailored” nanoparticles, and complemented the chemical conditions for the differential biosynthesis process. The present research represents a novel approach to manipulate cyanobacterial cells towards the production of different-sized gold nanoparticles whose curative impacts vary accordingly. This is the first report on that type of manipulated gold nanoparticles biosynthesis which will hopefully open doors for further investigations and biotechnological applications.

## 1. Introduction

*Cyanothece* is a spherical, unicellular cyanobacterial genus, with the capability of performing photosynthesis and nitrogen-fixation, despite their contradictory nature [1]. Photosynthesis generates oxygen, which is an inhibitor for the nitrogenase enzyme that is responsible for nitrogen fixation. However, this cyanobacterium is efficient in regulating those two contradictory processes through circadian rhythm, in which it performs photosynthesis during the day and nitrogen fixation during the night, thus contributing to nitrogen, oxygen, and carbon cycles. This remarkable metabolic capability was further confirmed through genomic studies [1]. *Cyanothece* sp. is a mixotroph, as it is capable of utilizing glycerol and glucose as energy sources in addition to performing photosynthesis to maximize its biomass yield [2].

On the medical and pharmaceutical frontiers, *Cyanothece* sp. was reported to contain sulphated polysaccharides that are capable of inhibiting the adhesion of pathogenic bacteria *Helicobacter pylori* to gastric epithelial cells [3]. Interestingly, extracts, derived from one *Cyanothece* sp. strain, had prominent anticancer impact on T-lymphoma cells, but not on myelogenic leukemia cells [4]. Moreover, the unicellular cyanobacterium was reported as a promising source of anticancer compounds [5] as its aqueous extracts were effective against cancer cell lines [6]. Despite their biotechnological importance and the diversity and impact of their unique bioactive compounds, many members of the *Cyanothece* genus have been scientifically underexplored [6]. 

Cyanobacteria are easily cultured in a cost-effective manner, both *in vitro* and *in vivo*, which makes them possible candidates for mass production of gold nanoparticles. However, some questions remain as to how to manipulate the size of biosynthesized nanoparticles and what are the conditions necessary for such manipulation. The main objective of the present study is to provide answers to those questions. Here, we focused on gold nanoparticles for the proposed customised nanoparticle biosynthesis. 

Gold is a biocompatible metal and, since ancient times, has been used as a drink for its curative properties for various diseases [7]. Gold nanoparticles (AuNPs) can be used in disease diagnosis and therapy, because they are biocompatible. In addition, these nanoparticles are advantageous in treatments for serious human diseases, such as HIV [7]. AuNPs can be biosynthesized by cyanobacteria, with noticeably different shapes and sizes among the species [7,8]. For instance, the cyanobacterium *Plectonema boryanum* biosynthesized octahedral AuNPs of 10 nm diameter. *Spirulina platensis* and *Calothrix* spp. produced spherical AuNPs of 20–30 nm diameter, whereas *Phormidium valderianum* biosynthesised bigger nanoparticles with hexagonal and triangular shapes. Another report on *Spirulina platensis* demonstrated that its AuNPs possessed spherical shape with a size range from 20–30 nm [9]. In our previous work, we demonstrated that the cyanobacterial strain, *Lyngbya majuscula,* biosynthesised spherical AuNPs of an average diameter of 41.7 ± 0.2 nm and those nanoparticles were proven to possess anti-myocardial infarction action [10].

Therefore, and in continuation of the series, this study came to investigate the ability of another type of cyanobacteria; the unicellular coccoid *Cyanothece* sp., to produce AuNPs. This study investigates the hypothesis of the capability of this coccoid cyanobacterium to produce different types or sizes of AuNPs under different chemical conditions and whether, or not, the change in the shape or size of the nanoparticles has an effect on myocardial infarction injury.

## 2. Results

### 2.1. Absorption and Luminescence Spectra and Percentage Yield of the Produced AuNPs

AuNP seed-treated cyanobacterial biomass started to develop light blue coloration both extra- and inter-cellularly within 60 min. The appearance of the blue color indicated the bioconversion of the ionic form gold (Au^3+)^ to the metallic form (Au^0^) and the formation of AuNPs. As time elapsed, the AuNP seed-treated cyanobacterial biomass turned dark blue. Meanwhile, the other cyanobacterial biomass where no AuNP seed solution was added, had turned purple. However, no change in color was observed in the control cyanobacterial cultures. The purple and blue colors were developed due to the reduction of gold salts using exopolysaccaharides [11] and were then stabilized by being linked to organic-sulphide [12,13] of the cyanobacterium. 

**Figure 1:**
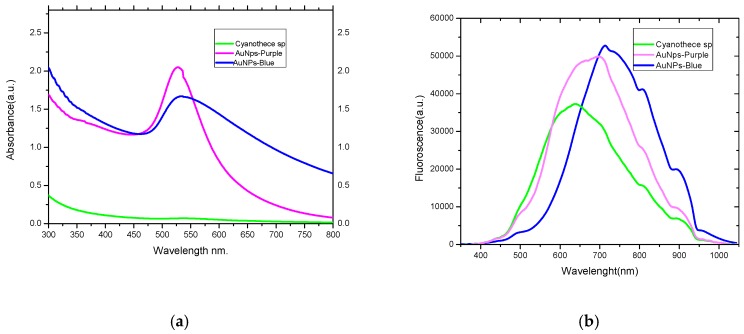
The UV-visible absorption (**a**) and the luminescence spectra (**b**) of control *Cyanothece* sp. cyanobacteria (green line) and gold nanoparticles produced by *Cyanothece* sp. (blue and purple color).

Figure 1 shows the surface plasmon resonance peaks (SPR) of the resultant purple and blue AuNPs that appeared at 535 and 525 nm, respectively. A gradual increase in purple and blue coloration in the cells indicated the steady synthesis of AuNPs which is most likely to be a function of time. The efficiency of production of AuNPs was calculated as 15% and 85% for the purple and blue AuNPs, respectively, through recording the percentage yield of the reduction process. The concentrations of purple and blueAuNPs were 2.5 and 25 µmole L^−1^, respectively, after 24 h.

### 2.2. Dynamic Light Scattering

Dynamic light scattering (DLS) was used to study the diameter, polydispersity, and concentration of the gold nanoparticles, (Table 1) DLS identified a polydisperse system of purple and blue AuNPs with particle sizes range of 80 ± 30 and 129 ± 40 nm in diameter, respectively. 

### 2.3. FTIR Analysis of Cyanothece sp and Its Complex with Gold Nanoparticles

**Figure 2:**
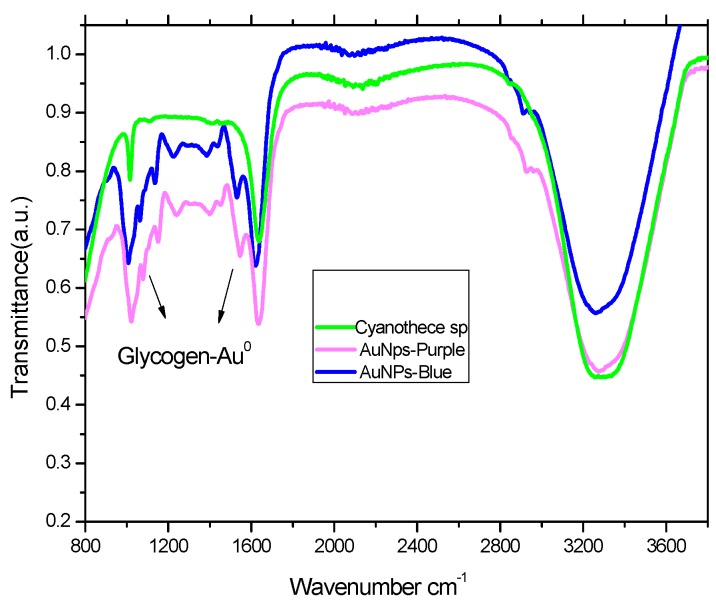
FTIR analysis of the gold nanoparticle complexes with *Cyanothece sp*.

A Fourier-transform infrared spectroscopy (FTIR) was applied to identify the glycogen content of *Cyanothece* sp., which was represented by absorption bands at 1000 to 1200 cm^−1^, ascribed for the AuNPs-glycogen complex [14], Figure 2. The absorption intensity of the purple-AuNPs-glycogen complex band was higher than that of the blue-AuNPs complex due to binding of the smaller purple nanoparticles to glycogen, which showed an increase in content, as indicated by the plasmon resonance peaks (SPR). It is most likely that glycogen plays a role as a nanoparticle stabilizer [15]. 

### 2.4. Cyclic-Voltammetry

**Figure 3:**
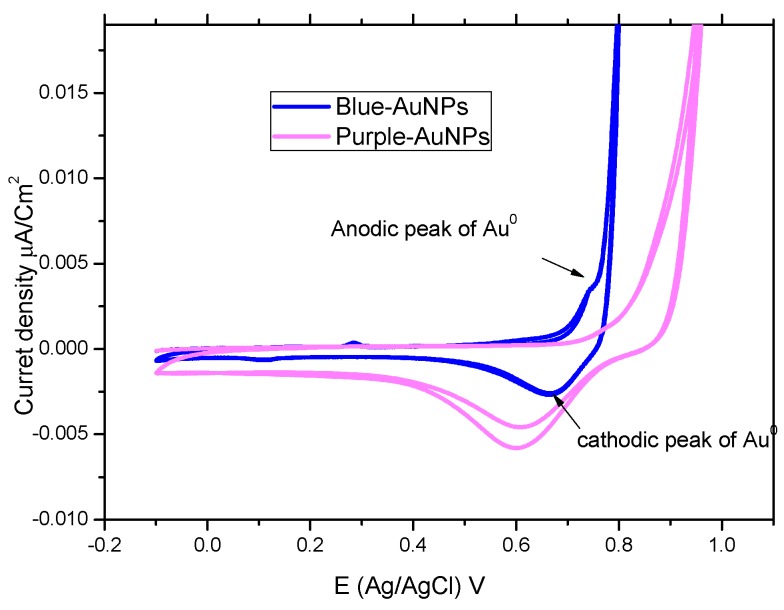
A cyclic voltammetry scan recorded for the cyanobacterial purple and blue AuNPs complexes at a scan rate of 100 mV/s.

Cyclic voltammograms (CV) for the cyanobacteria purple and blue-AuNPs complexes were recorded at the scan rate of 100 mV/s, as shown in Figure 3. blue-AuNPs have anodic and cathodic peaks around +0.74 V and +0.67 V (vs. Ag/AgCl), respectively, which reflect the oxidation of AuNPs and the subsequent reduction of the Au-oxide species back to AuNPs. The anodic peak of purple-AuNPs disappeared, and the cathodic peak was also shifted to less positive potential around +0.60 V (vs. Ag/AgCl). The disappearance of the anodic peak of purple-AuNPs is attributed to the rapid re-oxidation of AuNPs to Au-oxide ions in the absence of an exogenous capping agent [16].

### 2.5. Effect of Cyanobacterial Extract, AuNPs and their Combination on Cardiac Marker Enzymes

**Figure 4:**
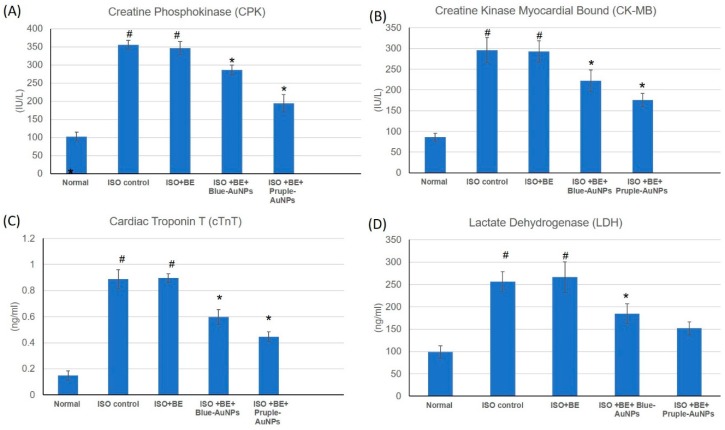
The treatment effects of bacterial extract (BE, 200 mg/kg/day, IP) and gold nanoparticles (blue-AuNPs or purple-AuNPs, 200 mg/kg/day, IP) combinations respectively, for 14 successive days on ISO-induced myocardial infarction with respect to serum level of cardiac marker enzymes. (**A**) creatine phosphokinase (CPK), (**B**) creatine Kinase-Myocardial Bound (CP-MB), (**C**) cardiac troponin T cTnT), and (**D**) lactate dehydrogenase (LDH) in normal and ISO-induced MI rats. Values were expressed as mean ± SD (n = 6). ISO: isoproterenol, BE: Cyanobacteria extract, AuNPs: gold nanoparticles, CPK: creatine phosphokinase, CK-MB: creatine kinase-myocardial bound, cTnT: cardiac troponin T, and LDH: lactate dehydrogenase. # indicates a statistically significant difference from the normal group, * indicates a statistically significant difference from the isoproterenol control group, (*p* < 0.05) using one-way ANOVA, followed by Tukey’s test as a post hoc analysis.

The effects of isoproterenol (ISO) and cyanobacterial extract (200 mg/kg/day, IP, BE), blue- and purple-AuNPs (20 mg/kg/day, IP, each), respectively, for 14 successive days and their combination at the mentioned doses on numerous cardiac indicator enzymes (Creatine Phosphokinase (CPK), Creatine Kinase-Myocardial Bound (CP-MB), Cardiac Troponin (T) (cTnT), and Lactate Dehydrogenase (LDH)) in normal and myocardial infarcted (induced via isoproterenol) tissues are illustrated in Figure 4. The cardiac indicator enzymes (CPK, CK-MB, cTnT, and LDH) were significantly augmented subsequent to isoproterenol administration and myocardial induction (Figure 4A–D) as compared with normal rats. Treatment with the combination of BE/ blue-AuNPs and purple-AuNPs with the mentioned doses, significantly improved the isoproterenol induced escalation of the diagnostic cardiac indicator enzymes (CPK, CK-MB, cTnT, and LDH), in comparison with isoproterenol-control rats. Furthermore, purple-AuNPs showed significant improvement in the results as compared to blue-AuNPs on CPK, CK-MB, and cTnT enzymes, but not on LDH. However, there were no significant alterations detected in the cardiac indicator enzymes in rats in the control or in bacterial extract (200 mg/kg/day, IP) alone.

### 2.6. Effect of Cyanobacterial Extract, AuNPs and Their Combination on Heart Rate and Blood Pressure Indices Recording and Measurement

**Figure 5:**
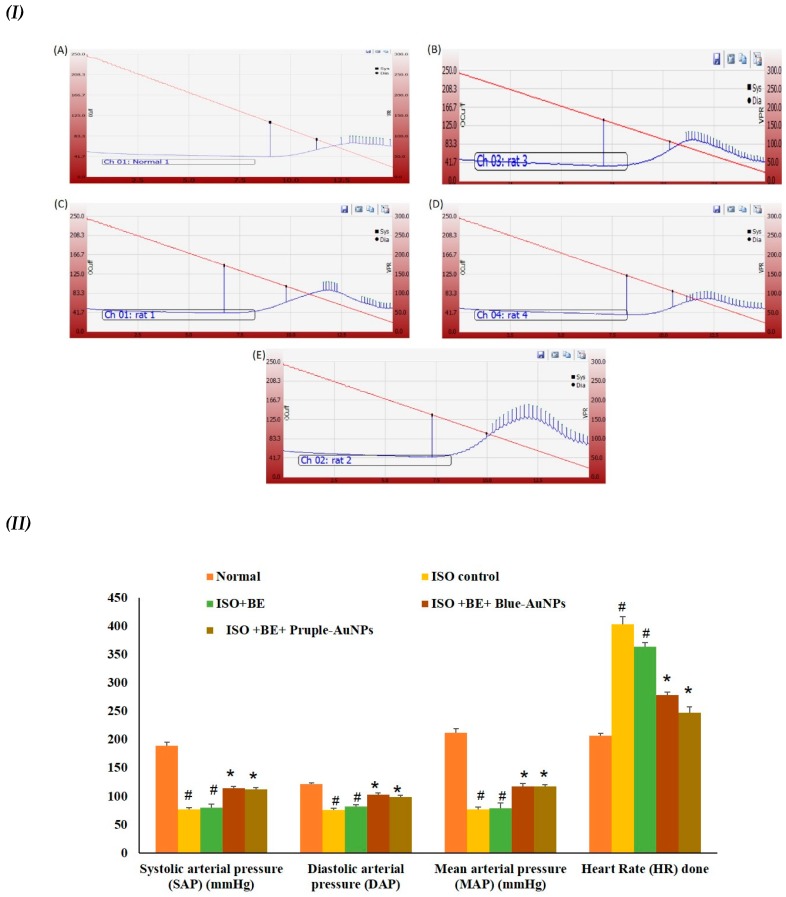
(**I**) Tracings obtained during blood pressure (BP) recordings. (**A**): Normal, (**B**): ISO control, (**C**): ISO + BE, (**D**): ISO +BE + blue-AuNPs, (**E**): ISO + BE + purple-AuNPs. (**II**) The treatment effects of bacterial extract (BE, 200 mg/kg/day, IP) and gold nanoparticles (blue-AuNPs or purple-AuNPs, 200 mg/kg/day, IP) combinations respectively, for 14 successive days on ISO-induced myocardial infarction (MI) with respect to SAP, DAP, MAP, and heart rate in normal and ISO-induced MI rats. All values were expressed as mean ± SD (n = 8).ISO: isoproterenol, SAP: systolic arterial pressure, DAP: diastolic arterial pressure, MAP: mean arterial pressure, and HR: heart rate. # indicates a statistically significant difference from the normal group, * indicates a statistically significant difference from the isoproterenol control group, (*p* < 0.05) group using one-way ANOVA, followed by Tukey’s test as a post hoc analysis.

The ISO injections induced a significant escalation in the heart rate and decline in systolic arterial pressure (SAP), diastolic arterial pressure (DAP), and mean arterial pressure (MAP) as compared to the normal animals group (Figure 5), indicating injury occurring in the myocardial tissues. However, the co-administration (BE + either blue-AuNPs or purple-AuNPs) with the mentioned doses significantly (*p* < 0.05) prohibited the ISO-induced deterioration in the arterial pressure indices SAP, DAP, and MAP. Similarly, the increase in heart rate (HR) was also attenuated in (BE + AuNP) rats as compared to the ISO control rats. However, there were no differences between the blue-AuNPs and purple-AuNPs on the arterial pressure indices or in the heart rate.

### 2.7. Effect of Cyanobacterial Extract, AuNPs and Their Combination on Electrocardiographic Trace Recording and Measurement

**Figure 6:**
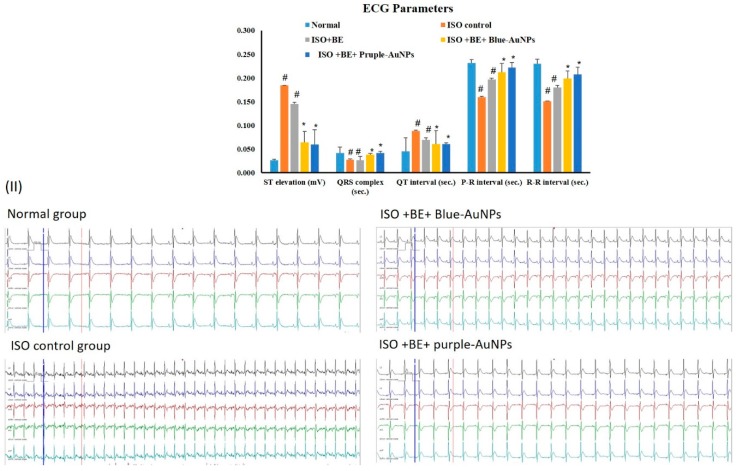
(**I**) The treatment effects of cyanobacterial extract (BE, 200 mg/kg/day, IP) and gold nanoparticles (blue-AuNPs or purple-AuNPs, 200 mg/kg/day, IP) combinations, respectively, for 14 successive days on isoproterenol-induced myocardial infarction with respect to the ECG components in normal and ISO-induced MI rats. All of the values are expressed as mean ± SD (n = 8). ISO: isoproterenol. # indicates a statistically significant difference from the normal group, * indicates a statistically significant difference from the isoproterenol control group, (*p* < 0.05) group using one-way ANOVA followed by Tukey’s test as a post hoc analysis. (**II**) Electrocardiogram (ECG) tracings that were recorded for evaluating heart rate and rhythm disorders.

Figure 6 shows a representative electrocardiographic trace of normal and experimental animals. Normal control, Control BE, Control AuNPs, and Control BE/ blue-AuNPs or purple-AuNPs -treated rats showed normal ECG patterns, whereas rats that were treated with isoproterenol showed a significant surge in the ST segment and QT intervals. Conversely, a significant decrease in QRS complex, and P-R and R-R intervals was seen in comparison with the control rats, which is indicative of infarcted myocardium. Treatment with blue-AuNPs and purple-AuNPs, respectively, for 14 successive days showed a reverse in ECG-induced alterations (Figure 6).

### 2.8. Effect of Cyanobacterial Extract, AuNPs and Their Combination on the Antioxidant Enzymes Activities in ISO-Induced MI in Rats 

**Figure 7:**
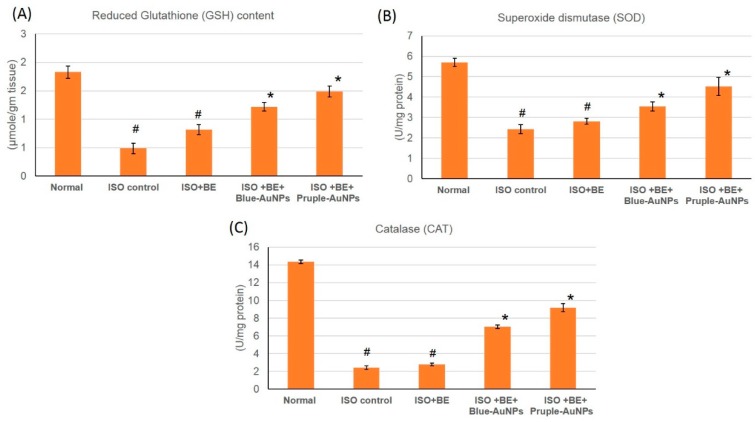
The treatment effects of cyanobacterial extract (BE, 200 mg/kg/day, IP) and gold nanoparticles (blue-AuNPs or purple-AuNPs, 200 mg/kg/day, IP) combinations respectively, for 14 successive days in isoproterenol (ISO)-induced myocardial infarction (MI) on (**A**) GSH, (**B**) SOD, and (C) CAT in normal and ISO-induced MI rats. All the values were expressed as mean ± SD (n = 8). ISO: isoproterenol, GSH: glutathione reductase, SOD: superoxide dismutase and CAT: Catalase. # indicates a statistically significant difference from the normal group, * indicates a statistically significant difference from the isoproterenol control group, (*p* < 0.05) using one-way ANOVA followed by Tukey’s test as a post hoc analysis.

The activities of the antioxidant enzymes GSH, SOD, and CAT were significantly decreased (*p* < 0.05) in the rats that were injected with ISO when compared to the control, which indicated a severe oxidative stress state existing within the myocardial tissue. However, treatment with blue-AuNPs or purple-AuNPs, 200 mg/kg/day, IP) respectively, for 14 successive days in rats with isoproterenol-induced myocardial infarction resulted in a significant increase in the activity of antioxidant enzymes GSH, and SOD when compared to the ISO-treatment alone (*p* < 0.05), Figure 7. In rats from the Control BE, Control AuNPs, and Control BE+ blue-AuNPs or purple-AuNPs groups, no significant differences in the activities of the antioxidant enzymes were detected when compared to the control.

## 3. Discussion

### 3.1. Metabolomic background of Cyanothece sp.

*Cyanothece sp.* is a genus of unicellular cyanobacterium that possesses the ability to fix nitrogen and perform photosynthesis. The cyanobacterium utilizes the daylight to generate high-energy carbohydrates, which are used at night to fix atmospheric nitrogen to produce ammonia, with hydrogen gas emerging as a by-product in a process catalyzed by the nitrogenase enzymes [17]. 

Chemically, *Cyanothece sp.* has a very simple metabolomic background, which is mainly composed of pigments, polysaccharides, fatty acids, polyphosphates, and minerals. *Cyanothece sp*. owes its blue-green color to a group of very special pigments called phycobiliproteins. These colored pigments aid in absorbing sunlight at wavelengths that chlorophyll does not absorb at. The colors of phycobiliproteins depend on the pigment phycobillin, which comprises an open chain tetrapyrrole structure covalently attached to a protein via the amino acid cysteine. These pigments can be classified according to their colors to phycocyanobilin (blue), phycoerythrobilin (red), and allophycocyanin (light-blue or violet) [18]. Commercially, these pigments are extensively used as fluorescent biological markers to label monoclonal antibodies and intact cells for diagnostic purposes. Additionally, they are used in food and beverage as colouring agents [19]. 

*Cyanothece sp.* has the ability to produce exopolysaccharides, a group of carbohydrates, which are normally secreted by microbial cells to act as a protective layer or a sheath [20]. The chemical composition of those polysaccharides differs according to the type of microbial organism and the environmental conditions. In an earlier study, *Cyanothece epiphytica* produced high concentrations of exopolysaccharides that included pentoses, hexoses, and deoxyhexoses in a polymerized structure through sulphate bonds in stressed conditions induced by ozone [20]. In another study, the *Cyanothece sp.* produced alpha-d-1,6-homoglucan as an exopolysaccharide [21]. Another important derivative of carbohydrates secreted by *Cyanothece sp.* is the sulphated polysaccharides which have multiple medicinal activities [22]. Other metabolites that were identified in the *Cyanothece sp.* include fatty acids, especially the C18 n-9 and n-3 fatty acids [17], polyhydroxyalkonoate and cyanophycin [12]. 

### 3.2. The Ability of Cyanothece sp. to Produce AuNPs 

This study investigated and confirmed the ability of *Cyanothece sp.* to reduce gold ions into AuNPs and to stabilize the produced nanoparticles. Several hypotheses were proposed to explain the mechanism of the bioconversion of Au^3+^ to AuNPs through the inter- and extracellular reduction of Au^3+^ to Au^0^. In one hypothesis, the reduction and the stabilization process can be attributed, at least in part, to the metabolomic background components of the cyanobacterium. Exopolysaccharides are suggested to contribute to the reduction of gold ions, both extracellularly and intracellularly [10]. The carboxylic groups in fatty acids and different protein moieties in the cell wall and membrane of the bacterium are believed to capture the gold ions for reduction. Furthermore, Patel et al. (2015) [23] reported that, the blue pigment phycocyanin can act as a stabilizer for nanoparticles. 

In another hypothesis, the electrostatic interactions make the gold ions adhere to the cyanobacteria outer cell wall, leading to an enzymatic reduction of gold ions, their aggregation, and finally AuNPs formation. [13]. A third hypothesis mentioned the binding of gold ions to sulfide residues of the sulphated polysaccharides of the bacterium sheath [12,13]. The microbial cell reduces metal ions by the use of specific surface reducing enzymes, such as NADH-dependent reductase or nitrate-dependent reductase [24,25]. 

In the present work, we suggest that the reduction of Au^3+^ to Au^0^ nanoparticles is biotically-mediated. The FTIR results indicated fluctuation in the glycogen level produced by the cyanobacteria according to the chemical environment used to produce AuNPs. Endogenous glycogen can act as an ‘electric wire’ or medium for electron transfer [26] which could facilitate the reduction process.

### 3.3. Cyanothece sp. Produces Two Types of AuNPs

The manipulation of cyanobacteria towards the differential biosynthesis of nanoparticles is a novel approach and it represented one of the main goals of the current research. Customized biosynthesis of AuNPs depend mainly on the chemical conditions in which the biosynthesis proceeds. Customization could be in the shape or size of the nanoparticles produced by the cyanobacterium. In the present investigation, and through controlling the chemical environment, *Cyanothece* sp. was able to produce two types of AuNPs; the purple and the blue nanoparticle, with an average diameter of 80 ± 0.3 and 129 ± 0.4 nm, respectively. 

The manipulation of the nanoparticles size was mainly mediated by the presence or absence of nanoparticles seeds. Other chemical factors which could attribute to such differentiation are the production of ammonia by the nitrogen-fixing ability of the cyanobacterium, rendered alkaline to the medium which favored the formation of blue-AuNPs [27]. Additionally, the fluctuation in glycogen level produced by the bacterium could be related to the type of nanoparticle synthesized, whether blue or purple. 

The size difference between the blue and purple AuNPs was confirmed by the SPR peaks which were visible between 529 and 536 nm, respectively, and it was previously confirmed that the SPR effect of gold nanoparticles is size-dependent [28,29]. The SPR peak of blue-AuNPs was shifted to longer wavelength due to the large diameter and non-spherical shape of the nanoparticles, while the purple AuNPs have high SPR due to the reactivity of smaller nanoparticles. The higher photoluminescence (PL) emissions of purple and blue-AuNPs nanoparticles around 695 and 718 nm are due to the surface plasmonic effect-collective oscillations of the 6sp band free electrons, as explained in Mie theory [30]. The higher PL emissions of blue-AuNPs are mostly related to the faster return of electrons to the ground state. 

SPR, diameter, concentration, and high mobility of gold nanoparticle electrons play a major role in medical treatments [27,30]. The smaller the nanoparticles, the larger their surface area, which enables them to come in contact with the biological cells, hence, smaller particles will have a higher percentage of interaction than bigger particles [31]. 

Dynamic light scattering (DLS) confirmed the chemical reactivity of purple and blue AuNPs, where light scattering was the highest for the blue-AuNPs due to the rapid Brownian motion and significant noise of laser light scattering, which could enable these nanoparticles to have better medicinal effect [32]. The appearance of anodic and cathodic peaks of blue-AuNPs in the cyclic voltammetry scan, reflects the oxidation of AuNPs and the subsequent reduction of the Au-oxide species back to AuNPs. Most of the Au^3+^ ions were bioconverted into Au^0^ nanoparticles in the blue-AuNPs products when compared to the reduction of Au-oxide to Au^0^ in the purple AuNPs [33]. 

### 3.4. The Two Forms of AuNPs Ameliorate the Myocardial Infarction State

Myocardial infarction (MI) is a leading cause of mortality worldwide according to numerous reports published by the American Heart Association [34]. MI is triggered by deficient myocardial oxygen supply as compared to demand, resulting in myocardial hypoxia and subsequent buildup of metabolites as waste. This is followed by several biochemical changes, such as free radical destruction, upsurge in cardiac indicators, and pro-inflammatory cytokines [35], oxidative stress, calcium overload, myocardial and endothelial injury, contractile dysfunction, necrosis, and /or apoptosis induced cell death [36]. Oxidative stress is one of the major mechanisms elaborated in myocardial infarction and cardiotoxicity pathogenesis. Isoproterenol administration produces a rapid, highly reproducible rat model of cardiac hypertrophy and myocardial infraction [7]. 

In our study, isoproterenol succeeded in the induction of the myocardial infarction condition in rats, as shown by elevated cardiac marker enzymes, irregularities in the heart rate, blood pressure, and ECG parameters, and the exhaustion of the anti-oxidant enzymes (GSH, SOD, and CAT). Treatment with cyanobacterial extract alone had no significant effect on the elevated cardiac marker enzymes, blood pressure and ECG parameters, or the anti-oxidant enzymes depletion. 

AuNPs have been produced by *Cyanothece sp.*, because of environmental incorporation of the gold ions by the cyanobacterium. The use of different sized AuNPs had achieved effective management of cardiac infarction, as can be seen from the adjustment of cardiac marker enzymes, reversal of ECG irregularities caused by isoproterenol, arterial pressure indices regularization, and the boost in antioxidant enzymes. These findings provided evidence that AuNPs could protect rat myocardium against ISO-induced myocardial injury and this protection could be attributed to the nanoparticle antioxidant activity [37,38]. 

Gold nanoparticles demonstrate relatively low cytotoxic and immunogenic activities [39], and especially the large ones (≥80 nm), show high biocompability and are non-cytotoxic to normal (non-cancerous) cell lines. These nanoparticles generally have high tolerability and a broad therapeutic index [40]. The cyanobacterium produced high levels of hydrogen as a result of the nitrogen-fixation process. These high levels of hydrogen synthesis can be proposed as a protection mechanism against oxidative stress, and thus could protect and even may treat the MI status [41,42]. 

Recently, the effect of progenitor (stem) cells in the repairing of tissue damaged in the MI status was illustrated and the influence of the cells was proven. The effect of plant extracts (such as date palm extract) that could accumulate in these cells (e.g., the CD34 and CD133 positive cells) in their reserve in the bone marrow and mobilized them toward the site of myocardial injury resulted in tissue repair and improvement of MI [43]. The effect of metal nanoparticles on the differentiation and mobilization of progenitor cells is reported [44,45]. Interestingly, the size of the metal nanoparticle was proved to change the rate of proliferation of some types of stem cell [41], which could explain the slight difference in between the purple (purple-AuNPs; 80 nm) and the blue (blue-AuNPs; 129 nm). This effect of AuNPs on the count and mobilization of progenitor cells needs further investigation and could be the topic for further studies. 

### 3.5. Conclusion

In summary, the manipulation of the cyanobacterium *Cyanothece* sp. towards the biosynthesis of different-sized gold nanoparticles was successfully achieved. The sulphated polysaccharides of this cyanobacterium, its unique physiological reductive abilities and enzymes as well as its stabilising entities were essential for nanogold biosynthesis. The latter biological attributes combined with the different specific chemical conditions allowed the customised nanogold biosynthesis. The curative effects of the different-sized nanoparticles were highly positive, with some differences mostly attributed to the difference in their cellular penetration abilities.

## 4. Materials and Methods

### 4.1. Isolation and Characterization of the Cyanobacterium Cyanothece sp. from the Arabian Gulf Region

**Figure 8:**
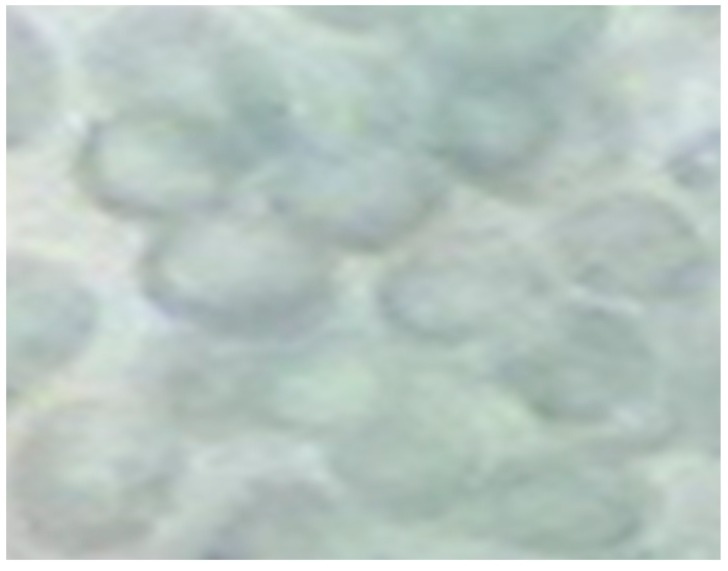
An image of the blue green cyanobacterium *Cyanothece* sp.

Water samples were collected from Al-Ahsa Governorate, Eastern Province, Kingdom of Saudi Arabia. Isolation of the cyanobacterial culture was achieved using F/2 medium [46], and characterization of the cyanobacterium as the coccoidal monocellular *Cyanothece sp.* as illustrated in Figure 8, was performed using light microscopy.

### 4.2. Production of Gold Nanoparticles in Cyanobacterial Cell Cultures 

Cyanobacterial cells were used for the bioconversion of Au^3+^ ion into blue and purple AuNPs according to Frens [47] with slight modifications. In brief, 900 μL of 25 mmol L^−1^ tri-sodium citrate was added to the solution containing 1 mL of 1 mmol L^−1^ HAuCl_4_ and 200 μL of 0.1 mol L^−1^ NaOH [48]. The reaction was allowed to proceed until the color became deep purple to form AuNP seed solution. To 5 mL of cyanobacterial culture, one ml of AuNP seed solution and 2 mL of 1 mmol L^−1^ HAuCl_4_ were added. The AuNPs agglomerated to form larger blue nanoparticles at room temperature after 60 min. In another experiment, the purple AuNPs were produced, in a similar manner by adding 3 mL of 1 mmol L^−1^ HAuCl_4_ to the solution of 5 mL cyanobacterial culture, however no AuNP seed was added. The gold nanoparticles solution turned purple after 24 hours. A control was included with both experiments where no HAuCl_4_ was added to the cyanobacterial cell cultures. 

### 4.3. Instrumental Analysis

#### 4.3.1. UV-Visible and Photo-Luminescence Spectroscopy and FTIR Analysis

The absorption and photo-luminescence spectra were recorded on spectrophotometer and spectrofluorometer models USB4000 and USB4000FL (Ocean Optics Devices, Winter park, FL, USA), respectively. A FT-IR spectrophotometer (Bruker Alpha, Billerica, MA, USA) was used to study the absorption spectra of the cyanobacteria alone and with AuNPs complexes.

#### 4.3.2. Dynamic Light Scattering

The non-invasive dynamic light scattering technique was applied to measure the size distribution of AuNPs suspensions. The dynamic light scatter (DLS) instrument model Nano DS, SN 161 was used to determine the hydrodynamic radius, polydispersity system and the concentration of AuNPs.

#### 4.3.3. Cyclic Voltammetry

Electrochemical analyses were performed using a Nuvant EZstat Pro potentiostat in a three-electrode system with a standard Ag/AgCl reference electrode (prepared in saturated KCl solution), Pt-wire counter electrode, and gold working electrode (diameter, 2 mm). The cyanobacteria and cyanobacteria-AuNPs complex samples were measured at the scan rate of 100 mV·S^−1^ without pH adjustment or supporting electrolyte due to the salt-containing nature of the samples [49].

#### 4.3.4. Calculation of AuNPs Efficiency of Production

The efficiency of production of AuNPs (as a percentage) was calculated after 24 hours using the following equation [13]:

The percentage of yield = AuNPs con./Gold salt con.*100

Concentration unit, µmol L^−1^

### 4.4. Determination of the Anti-Myocardial Infarction Activity

#### 4.4.1. Materials

Isoproterenol (ISO) (Cat. No I6504), and Creatine Phosphokinase (CPK) ELISA kits (Cat. No. C3755-3.5KU) were purchased from Sigma-Aldrich (St. Louis, MI, USA). Cardiac Tropinine T (cTnT) ELISA kits (Cat. No. mbs162871) were bought from My BioSource, Santiago, CA, USA, while the Creatine Kinase—Myocardial Bound (CK-MB) ELISA (Cat. No. ABIN955837), Glutathione (GSH) assay (ab102530) and Superoxide Dismutase (SOD) activity assay (ab65354) kits were obtained from Abcam Co., Ltd. (Hong Kong, China). The Catalase (Cat. No. 707002) and Lactate Dehydrogenase (LDH) Assay kits (Cat. No 601170) were acquired from Cayman Chemical, Michigan, USA. Further chemicals and reagents used were of the highest analytical grade and acquired from commercial sources.

#### 4.4.2. Extraction of the *Cyanothece sp.*

Sixty day-old cultures of *Cyanothece* sp. were evaporated under reduced pressure (temperature did not exceed 35 °C and the pressure was 100 mbar) and then the residue was freeze-dried. The powder produced from the freeze-drying process was subjected to extraction with 100% ethanol (100 mL/one gram powder) three times and the ethanol extracts were filtered and collected. The ethanol extract was evaporated under reduced pressure and the residue was dissolved in DMSO to give 200 mg/mL stock solution. 

#### 4.4.3. Preparation of AuNPs Solution for Animal Application

The gold concentration of freshly filtered AuNPs was determined using the particle diameter and absorbance. The concentration of AuNPs solution was adjusted to give a stock solution of 200 mg/mL

#### 4.4.4. Animals

Sprague–Dawley male rats (170 ± 25 g) were acquired from the animal house facility, King Saud University, Riyadh, kept in standard laboratory conditions (23 °C ± 1 °C), and maintained on a standard commercial rodent diet using a 12 h light/dark cycle during the accommodation period. The Animal Research Ethics Committee at King Faisal University approved all of the animal experimental procedures and protocols, and they were performed in accordance with the Guidelines for the Ethical Conduct for Use of Animals in Research, King Faisal University.

#### 4.4.5. Experimental Design

The rats were allocated into eight groups (six rats per group, n = 6) after acclimating to the facility. Groups 1–4 were given SC (saline injection), while groups 5–8 were injected with isoproterenol (100 mg/kg, SC). The injections were given once a day for three days in both sets. Groups 1 and 2 were considered as control groups (Normal and Control Bacterial extract BE) following the three-day injection period, and were injected with normal saline (IP) and *Cyanothece sp* extract (200 mg/kg/day, IP) respectively for 14 successive days. Groups 3 and 4 were given blue-AuNPs and purple-AuNPs (200 mg/kg/day each, IP) for 14 successive days (Control AuNPs groups). Groups 5 was treated as an isoproterenol control group (ISO control) and was injected with normal saline (IP) for 14 successive days. Groups 6 was given *Cyanothece sp* extract (200 mg/kg/day, IP), for 14 successive days, respectively. Groups 7 and 8 were given a mixture of *Cyanothece sp* and blue-AuNP and purple-AuNP (200 mg/kg/day, each, IP) for 14 successive days (BE + AuNPs groups).

#### 4.4.6. Electrocardiogram (ECG) and Blood Pressure (BP) Recording and Measurement

ECG recordings were performed on urethane-anesthetized rats (1.5 g/kg), after 14 successive days, using a noninvasive computerized ECG apparatus from Kent Scientific (Torrington, CT, USA). Heart rate, RR and QT intervals, R wave amplitude, and ST segments were calculated from ECG recordings by computer. BP measurements were obtained using a noninvasive, computerized tail-cuff system from Emka Technologies’ systems (Paris, France).

#### 4.4.7. Tissue Handling and Biochemical Estimation

Blood samples were collected and centrifuged (10 min/4000 rpm) to separate serum, which was then stored at −80 °C prior to analysis. Anesthetized animals were then sacrificed by cervical dislocation. For biochemical parameters, heart tissue samples were homogenized using phosphate buffer saline (50 mmol L^−1^, pH 7.4). The acquired homogenates (10% w/v) of different investigational groups were centrifuged (12,000 rpm/20 min/4 °C), and the collected supernatants were used for the determination of different biochemical parameters.

#### 4.4.8. Determination of Cardiac Marker Enzymes

ELISA kits, following the manufacturer’s instructions, were used to measure the serum levels of Creatine Phosphokinase (CPK), Cardiac Tropinine T (cTnT), Creatine Kinase—Myocardial Bound (CK-MB) and Lactate Dehydrogenase (LDH).

#### 4.4.9. Estimation of Antioxidant Activity

Heart tissue supernatants were used to evaluate the endogenous antioxidative enzyme activities, comprising reduced Glutathione (GSH), Superoxide Dismutase (SOD), and Catalase (CAT), using standard assay kits and a microplate reader (VERSAmax™, Molecular devices, Sunnyvale, CA, USA).

### 4.5. Statistical Analysis

All of the values were expressed as mean ± SEM (n = 6). Image analysis was completed using Image J software. Graph Pad Prism 5 software was performed to evaluate the statistical analysis. The value *p* < 0.05 was considered to be statistically significant using one-way analysis of variance (ANOVA), followed by Tukey’s test.

## Figures and Tables

**Table 1:** The properties and diameter of gold nanoparticles (AuNPs) produced by culture of the cyanobacterium *Cyanothece sp.*

Sample	Diameter (nm)	Aggregation Time	Concentration (µmole.L^−1^)	Shape	System
blue- AuNPs	129 ± 0.4	60 min	25.00	Non-spherical	Polydisperse
purple-AuNPs	80 ± 0.3	24 h	2.50	Spherical	Polydisperse
